# Formulation and clinical translation of inhalable nanomedicines for the treatment and prevention of pulmonary infectious diseases

**DOI:** 10.1007/s13346-025-01861-5

**Published:** 2025-04-29

**Authors:** Rami Ahmed, Frederic Tewes, Marique Aucamp, Admire Dube

**Affiliations:** 1 School of Pharmacy, University of the Western Cape, Robert Sobukwe Road, Bellville, 7535 Cape Town South Africa; 2 INSERM U1070, Pôle Biologie-Santé – B36, 1 Rue Georges Bonnet, 51106, 86073 POITIERS Cedex 9, TSA France

**Keywords:** Nanoparticle pulmonary drug delivery, Clinical translation of inhaled nanomedicines, Infectious diseases and nanomedicine, Inhalable nanoparticle

## Abstract

Pulmonary infections caused by bacteria, viruses and fungi are a significant global health issue. Inhalation therapies are gaining interest as an effective approach to directly target infected lung sites and nanoparticle-based pulmonary delivery systems are increasingly investigated for this purpose. In this review, we provide an overview of common pulmonary infectious diseases and review recent work on the application of inhalable nanoparticle-based formulations for pulmonary infectious diseases, the formulation strategies, and the current research for delivering inhalable nanomedicines. We also evaluate the current clinical development status, market landscape, and discuss challenges that impede clinical translation and propose solutions to overcome these obstacles, highlighting promising opportunities for future advancements in the field. Despite advancements made and products reaching the market, notable gap persists in translational research, with challenges in achieving the target product profile, availability of appropriate in vivo disease models, scale-up, and market related questions, likely hindering research translation to the clinic.

**Graphical Abstract:**

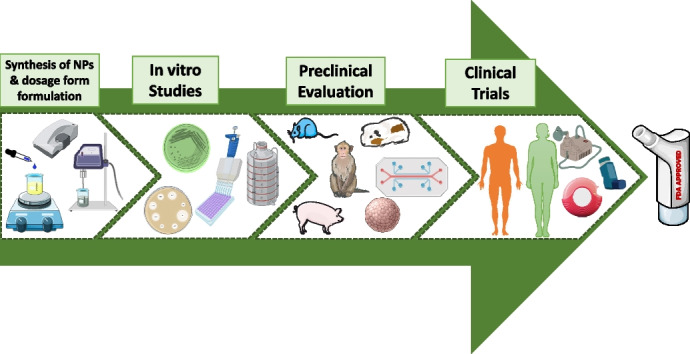

## Background

Infectious diseases are illnesses that are transmitted from person to person, or between animals and humans. These diseases often spread through actions such as coughing, sneezing, or sharing of bodily fluids. Pathogens involved in the disease can be transmitted through direct human contact, interactions with animals, or through vectors such as insects [1]. The lungs, as the primary organ of the respiratory system, are continually exposed to harmful agents in the air, including pollutants and hazardous materials and hence also vulnerable to infections caused by various pathogens, including viruses, bacteria, and fungi. Common lung infections, such as pneumonia, tuberculosis (TB), and bacterial diseases associated with chronic obstructive pulmonary diseases (COPD) and cystic fibrosis (CF), are often life-threatening [2]. These infections are becoming increasingly challenging to treat and eradicate due to rising antibiotic resistance and a thin pipeline of new antimicrobials [3]. More than 200 infectious diseases have been identified, and outbreaks are a regular occurrence. Table 1 outlines common pulmonary infectious diseases and the responsible pathogens.

**Table 1:** Outline of common pulmonary infectious diseases and the responsible pathogens

Microorganism type	Disease	Pathogen	Region of pulmonary tract infection	Reference
**Viral infection**	Avian flu	*Influenza A(H5 N1, H5 N6, H3 N8, H7 N4, H7 N9, H9 N2, and H10 N3) viruses*	Lower respiratory tract (bronchi, bronchioles, and alveoli)	[4]
	Influenza (Flu)	*Influenza viruses (Type A, B, and C)*	Upper (nose and throat) and lower (trachea, bronchi, bronchioles, and alveoli) respiratory tract	[5]
	Pharyngitis, tracheobronchitis, and pneumonia	*Human Parainfluenza virus*	Upper (nose and throat) and lower (trachea, bronchi, bronchioles, and alveoli) respiratory tract	[6]
	Common cold	*Rhinovirus/Enterovirus*	Upper (nose and throat) and lower (trachea, bronchi, bronchioles, and alveoli) respiratory tract	[7]
	Acute Bronchitis and pneumonia	*Adenovirus*	Upper (nose and throat) and lower (trachea, bronchi, bronchioles, and alveoli) respiratory tract	[8, 9]
	COVID- 19 (Acute bronchitis and pneumonia)	*SARS-CoV- 2 virus*	Upper (nose and throat) and lower (trachea, bronchi, bronchioles, and alveoli) respiratory tract	[10]
	Pharyngitis, Laryngotracheitis, and pneumonia	*Herpes simplex/herpes zoster virus*	Lower respiratory tract (trachea, bronchi, and alveoli)	[11]
	Viral Pneumonia	*Rubeola virus*	Alveoli and bronchial airways	[11]
		*Cytomegalovirus*	Alveoli and interstitial tissue of the lungs	
	Viral pneumonia and bronchiolitis	*Respiratory syncytial virus (RSV)*	Upper (nose and throat) and lower (trachea, bronchi, bronchioles, and alveoli) respiratory tract	[12]
**Bacterial infection**	Bacterial pneumonia	*Streptococcus pneumoniae*, *Haemophilus influenzae*, *Staphylococcus aureus (SA), methicillin resistant SA (MRSA), or Klebsiella pneumoniae*	Lower respiratory tract (bronchi, bronchioles, alveoli, and lung parenchyma)	[13]
	Pulmonary nocardiosis	*Nocardia (Nocardia Asteroids)*	Lower respiratory tract (bronchi, bronchioles, alveoli, lung parenchyma, and pleura)	[14]
	Tuberculosis	*Mycobacterium tuberculosis (M. tb)*	Lower respiratory tract (bronchi, alveoli, pleura, and upper lobes of the lungs)	[15]
	Nontuberculous mycobacterial lung disease	*Mycobacterium avium complex (MAC)*	Lower respiratory tract (bronchi, bronchioles, alveoli, and lung parenchyma)	[16]
	Legionnaire’s disease	*Legionella spp*	Lower respiratory tract (bronchi, bronchioles, alveoli, and lung parenchyma)	[11]
	Pulmonary actinomycosis	*Actinomyces spp*	Lower respiratory tract (bronchi, bronchioles, alveoli, lung parenchyma, pleura, and chest wall)	[17]
	Bacterial infections associated with chronic obstructive cystic fibrosis (CF)	*Pseudomonas aeruginosa, Achromobacter xylosoxidans, and Stenotrophomonas maltophilia*	Lower respiratory tract (bronchi, bronchioles, alveoli, and lung parenchyma)	[18, 19]
	Pneumonic plague	*Yersinia pestis*	Lower respiratory tract (bronchi, bronchioles, and alveoli)	[20, 21]
**Fungal infection**	Histoplasmosis	*Histoplasma capsulatum*	Lower respiratory tract (bronchi, bronchioles, alveoli, lung parenchyma, and lymph nodes)	[11, 22]
	Pulmonary cryptococcosis	*Cryptococcus neoformans*	Lower respiratory tract (bronchi, bronchioles, alveoli, and interstitial lung tissues)	[23]
	Coccidioidomycosis	*Coccidioides immitis*	Lower respiratory tract (bronchi, bronchioles, alveoli, interstitial lung tissues, and pleura)	[22]
	Pulmonary Aspergillosis	*Aspergillus* spp	Lower respiratory tract (bronchi, bronchioles, alveoli, and lung parenchyma)	[24]
	Pulmonary candidiasis	*Candida spp*	Lower respiratory tract (bronchi, bronchioles, alveoli, and lung parenchyma)	[22]

The COVID- 19 pandemic, caused by the SARS-CoV- 2 virus rapidly spread globally via human interaction, leading to a worldwide health crisis with significant mortality and profound social and economic impact. According to data from Johns Hopkins University Coronavirus Resource Center, by October 2023, there were over 660 million confirmed COVID- 19 cases globally and more than 6.5 million deaths worldwide [25, 26]. Another notable viral outbreak is the avian flu, which emerged from Asia [27, 28]. According to the WHO report, as of January 2024, there have been approximately 2,000 confirmed cases of human infections with avian influenza, leading to a total of 793 deaths​ [29].

Oral drug administration is widely employed for systemic treatment, including for pulmonary infections. Its effectiveness is attributed to the relatively large absorption capacity of the intestine, combined with convenience and patient acceptability of this route, which promotes better adherence [30]. However, many antimicrobials used for pulmonary infections are classified as Biopharmaceutical Classification System (BCS) Class II or IV drugs [31–33]. The low solubility hinders absorption in the gastrointestinal tract, leading to reduced bioavailability [34]. Additionally, oral antimicrobials encounter challenges such as enzymatic and/or pH-mediated degradation, poor specificity to target sites, and potential toxicity due to nonselective drug distribution [35, 36].

The inhalation route provides a number of benefits compared to oral or even intravenous administration. Delivery of therapeutics directly to the lungs for local action is advantageous due to the unique anatomical and physiological features of the lung [37]. These benefits are established and include the extensive surface area of the alveolar epithelium, comparatively lower local enzymatic activity than that of the gastrointestinal tract, and bypassing hepatic first-pass metabolism [38–40]. ​For low-potency drugs like antimicrobials, achieving therapeutic efficacy via inhalation may necessitate large doses, potentially exceeding tolerability limits [41]. However, targeted delivery can concentrate high drug levels at the infection site while minimizing systemic exposure [38–40]. This approach enhances efficacy and reduces systemic off-target effects. Despite the advantages of inhalation therapy, this route faces challenges such as the rapid clearance of particulate drug delivery systems by alveolar macrophages, which reduces their residence time and concentration near the pathogens [42]. Furthermore, issues such as inefficient drug deposition in the bronchial tree, potential degradation of drugs, and non-selective targeting may require higher doses, raise concerns about toxicity [43]. Lung epithelium also lacks specialized transporters and channels present in the liver and intestine, hindering effective drug absorption and distribution within the lungs. On the other hand, directly targeting alveolar macrophages holds significant potential to enhance the effectiveness of therapies for diseases where pathogens reside within macrophages such as TB [44–48].

Therapeutic effectiveness, patient adherence, and market success largely depend on the delivery method [49]. Nanoparticles (NPs) enhance drug uptake across biological barriers, enable controlled release, and facilitate site-specific, targeted intracellular delivery, allowing for a reduced dose to achieve the desired therapeutic effect [47, 50, 51]. NPs can be tailored to overcome common physiological barriers and reduce rapid clearance from the respiratory tract, making them suitable for treatment of pulmonary infectious diseases [25, 52]. Approaches often involve modifying key physicochemical properties such as size, shape, surface charge, and lipid solubility, or conjugating the NPs with targeting moieties to enhance lung epithelium penetration and achieve cellular and intracellular organelle targeting [53–55]. Various techniques have been explored to formulate NPs within a respirable particle size range (1–5 µm) to ensure effective delivery to specific lung regions. Inhalation devices, such as nebulizers, play a critical role in optimizing lung deposition by controlling droplet size [56]. NPs are incorporated into micron-sized droplets delivered by nebulizers, ensuring efficient pulmonary delivery [56]. In the case of inhalable dry powders, NPs can be adsorbed onto carriers or formed into weakly agglomerated structures that dissociate or fragment easily upon contact with lung fluids, enhancing effective delivery and ensuring optimal deposition in the desired regions of the respiratory tract [57].

In this paper, we review studies to address lung infections using NP-based formulations. We discuss strategies for formulating inhalable products and evaluate the current clinical status and market landscape. Additionally, we discuss the limitations of clinical translation, propose approaches to overcome these challenges towards clinical translation of more of these medicines.

## NPs for pulmonary delivery of therapeutics

Liposomes, lipid and polymeric NPs have been most commonly investigated for inhalation, likely due to their biocompatibility. Our analysis, using data from PubMed, [58], shows that by December 2024, there were 53,187 publications on nanomedicines, including 23,355 on infections, and 3,373 specifically focused on inhalable NPs, with only 502 articles combining both inhalable NPs and infections. Table 2 summarizes the main NP-based formulations for pulmonary delivery in the context of infectious diseases. The table highlights the primary components and properties of the NPs, including aerodynamic properties following formulation as nebulization/aerosol or after being loaded in an inert carrier in the case of inhalable powders and the therapeutic agents delivered. Additionally, it summarizes the models used for evaluating these nanomedicines and the observed outcomes.

**Table 2:** Summary of studies on the application of nanoparticle-based formulations for treatment and prevention of pulmonary infectious diseases

Indication/purpose	NPs type	Therapeutics agent	Particle size and aerodynamic properties	In vitro/in vivo model	Method of administration to the lungs	Functions/observations	Reference
**Pulmonary tuberculosis**	Liposomes modified with phosphatidylserine (PS) and phosphatidic acid (PA)	–	Particle size = 1–2.5 µm	**In vitro***:* THP- 1, M1 or M2 macrophages, A549 cell **In vivo***:* BALB/c mice	Intranasal	These liposomes featured PS on their outer surface to promote phagocytic uptake and PA on the inside to facilitate phagolysosome maturation. This design ensured effective macrophage uptake and improved intracellular killing of *M.tb*	[90]
	Liposome	Levofloxacin	NP size ≥ 200 nm MMAD = 5.9 μm FPF = 60%	**In vitro***:* THP- 1 cells **In vivo:** N/A	DPI	Showed good aerodynamic properties, biocompatibility, and improved macrophage uptake, which were further enhanced after spray drying	[67]
	Liposome-mannose	Moxifloxacin	NP size = 372–379 nm MMAD = 1.47 − 4.76 μm FPF = 66.6–72.1%	**In vitro***:* A549 cells, J774 macrophages **In vivo:** rats	DPI	**In vitro*:*** The nanoformulation demonstrated higher antitubercular activity against *M. tb* and improved macrophage uptake. **In vivo:** It showed localization primarily in the alveolar spaces, with minimal or very low deposition in the upper respiratory tract	[65]
	SLNPs modified with mannose	Rifampicin	MMAD = 0.72–1.38 μm The respirable fraction is 2.0–25.3%	**In vitro:** J774 macrophages cells **In vivo:** N/A	DPI	Physical characteristics appropriate for targeting of alveolar macrophages	[97]
	SLNPs modified with mannose	Rifampicin	NP size = 309 nm – 740 nm FPF = 11.77–53% An aerodynamic diameter = 210—676 nm	**In vitro:** MH-S cell line **In vivo:** N/A	DPI	SLNPs were inhalable and effectively targeted alveolar macrophages	[69]
	SLNPs	Rifampicin, isoniazid and pyrazinamide	MMAD = 1.7 µm GSD = 1.9 µm	**In vitro:** N/A **In vivo:** guinea pigs	Nebulization	The NPs enhance drug bioavailability and decrease dosing frequency, leading to improved management of pulmonary tuberculosis	[98]
	PLGA- poly-L-lysine (PLGA-PLL)	Rifampicin	NP size = 0.5–2 μm	**In vitro*:*** THP- 1 macrophages and murine primary bone marrow derived macrophages **In vivo:** Mice	Intratracheal (powder)	PLGA-PLL shows greater macrophage uptake both in vitro and in vivo compared to non-modified particles	[77]
	β-glucan-chitosan-PLGA	Rifampicin	NP size = 235 nm	**In vitro*:*** N/A **In vivo:** Mice	Oropharyngeal aspiration and oral gavage	Pharmacokinetic studies indicate that rifampin maintains a sustained release in vivo for more than a week. Additionally, the analysis reveals activation of the innate immune system, as shown by cytokine profiling	[51]
	Curdlan-PLGA	-	NP size = 235—464 nm	**In vitro*:*** RAW264.7 cells **In vivo:** Mice	Oropharyngeal aspiration	The curdlan–PLGA NPs induced autophagy in *M. tb*-infected macrophages, reduced *M. tb* lung burden, and modulated immune responses in infected mice	[85]
	Alginate	Isoniazid, rifampicin and pyrazinamide	NP size = 235.5 nm MMAD = 1.1 μm GSD = 1.71 μm	**In vitro*:*** N/A **In vivo:** Guinea pigs	Intranasal	Encapsulating drugs in alginate NPs greatly improved their bioavailability compared to free drugs. The encapsulated drugs remained above the MIC in organs for up to 15 days after nebulization, while free drugs were only detectable for one day	[99]
	Polymeric micelles	Rifampicin	NP size = 107 nm MMAD = 3.86 μm FPF o = 57%	**In vitro*:*** THP- 1 macrophages, **In vivo:** Rats	Suspension for nebulization	The NPs increased microbicidal activity against *M. tb*-infected THP- 1 macrophages by up to 2.5 times compared to rifampicin solution and showed sustained lung accumulation for 24 h in rats	[100]
	Polymeric micelles -mannose	Rifampicin-curcumin	NP size = 80—366 nm MMAD = 1.26 − 2.52 μm FPF = 19–75%	**In vitro*:*** THP- 1 macrophages **In vivo:** Rats	Suspension for nebulization	The nanoformulation's aerodynamic diameter allows it to reach the deep lung, where biodistribution studies confirmed sustained lung accumulation over 24 h. Mannose coating also significantly improved in vitro microbicidal efficacy against *M. tb* H37Rv	[101]
	PAMAM dendrimer	Rifampicin	MMAD = 2–6 µm FPF = 42–54%	**In vitro*:*** N/A **In vivo:** Wister rats	DPI using microsprayer syringe	In vivo studies showed that intratracheal administration of these particles can reach the MIC of rifampicin in plasma	[102]
**Nontuberculous mycobacterial lung disease (*MAC*)**	Liposomes- phosphatidylcholine-cholesterol	Rifampicin	NP size = 180–220 nm All formulations had a nebulization efficiency > 50%	**In vitro***:* J774 macrophages cells **In vivo**: Wistar albino rats	Suspension for nebulization	Liposomes proved effective against *MAC* in infected macrophages in vitro and successfully reached the lower airways in rats	[66]
**Pulmonary bacterial infection *Mycobacterium smegmatis (M. smeg)***	Liposomes-phosphatidylcholine-cholesterol- modified with, dicetylphosphate (DCP) and O-steroyl amylopectin (O-SAP)	Rifampicin	MMAD = 2.32–3.85 μm Each actuation released 101–113 mg of liposomal suspension	**In vitro***:* N/A **In vivo***:* Albino wistar rats	Pressurized aerosol (liposomal suspension)	Ligand-anchored liposomal aerosols significantly reduced *M. smeg* viability inside macrophages (in vitro*)* to 7–11% after administration of drug *(*in vivo) and effectively maintained high drug concentrations in lung regions rich in alveolar macrophages	[68]
	Amphiphilic lipopolymer (stearic acid (SA) and branched polyethyleneimine (BPEI)	Rifampicin	NP size = 0.22–1.38 μm MMAD = 2.3–2.5 μm FPF = 67.88%	**In vitro*:*** THP- 1 cells **In vivo:** N/A	DPI	A biocompatible formulation with an initial burst release, followed by sustained release over 24 h, was developed. Additionally, the NPs showed colocalization with *M. Smeg* inside THP- 1 cells, making them suitable for targeting *M. tb* within the phagosomes of alveolar macrophages	[103]
**Pulmonary bacterial infection (intracellular parasites in alveolar macrophages)**	Liposomes-mannose	Ciprofloxacin (CPX)	NP size = 1 µm	**In vitro***:* N/A **In vivo***:* Rat	Suspension for nebulization	Mannosylated CPX-liposomes showed strong antibacterial activity against various bacteria, unlike unmodified CPX-liposomes, and had a low risk of microbial mutation	[104]
**Pulmonary bacterial infection* (P. aeruginosa* and *S. aureus)***	SLNPs-chitosan-eugenol (SLNPs/Chi/Eu)	Ofloxacin (Ofx)	NP size = 128–304 nm	**In vitro*:*** A549 and Wi- 38 **In vivo:** mice	DPI	**In vitro*:*** The SLN were nontoxic and demonstrated enhanced bactericidal activity against *P. aeruginosa* and *S. aureus*, with the MIC for Ofx formulations dropping by 6.1- to 16.1-fold when encapsulated in SLN/Chi/Eu **In vivo*:*** effectively delivered therapeutic doses of Ofx to mouse lung via nasal administration	[105]
**Pulmonary bacterial infection (*P. aeruginosa*, *K. pneumoniae*, and *S. pneumoniae*)**	Niosomes, Span 60, Tween 60, cholesterol	Ciprofloxacin (CPX)	MMAD = 4.21–8.79 μm FPF = 50.5–68.3%	**In vitro*:*** A549 cell **In vivo:** N/A	Nebulization using air-jet nebulizer	Niosomal CPX had a lower MIC against *P. aeruginosa*, *K. pneumoniae*, and *S. pneumoniae* compared to free CPX, with significantly lower cytotoxicity in the A549 cell line and favourable aerodynamic properties	[106]
**Pulmonary bacterial infection* (S. aureus s and M. abscessus)***	PLGA and chitosan	Clarithromycin	NP size = 94—120 nm	**In vitro*:*** RAW264.7 cells, murine wound model **In vivo:** zebrafish	Suspension for nebulization	Nanocapsules loaded with the drug were significantly more effective at killing intracellular *S. aureus* and both *M. abscessus* variants compared to the same dose of the free drug	[107]
**Pulmonary bacterial infection (*P. aeruginosa, K. pneumoniae, and Acinetobacter baumannii*)**	PLGA-hyaluronic acid	Polymyxin B (PMB)	NP size = 170–220 nm	**In vitro*:*** N/A **In vivo:** Mice	Nebulization	NPs outperformed free PMB in mucus penetration and alveolar cell delivery, showed better biocompatibility with reduced toxicity, and offered superior antimicrobial efficacy in treating lung infections in mice	[108]
**Pulmonary bacterial infection* (P. aeruginosa)***	PLGA	Esculentin- 1a-Derived Peptide	NP size = 261–282 nm MMAD = 2.66–3.89 μm FPF = 27.6–41%	**In vitro*:*** N/A **In vivo:** Mice	Liquid jet nebulizer	Peptide-loaded NPs effectively inhibited *P. aeruginosa* growth *(*in vitro) and achieved a 3-log reduction in pulmonary bacterial burden for up to 36 h following a single intratracheal administration (in vivo)	[109]
	PLGA optimized either with PVA, chitosan or alginate	Tobramycin	NP size = 250 nm MMAD = 3.3–3.4 μm FPF = 52%	**In vitro*:*** A549 cell **In vivo:** Wistar rat	DPI-Nano-embedded micro-particles intra-tracheally	Optimized formulations showed strong in vitro antimicrobial activity against *P. aeruginosa*, with NP composition crucial for determining the in vivo deposition pattern of nanoembedded microparticle	[110]
	Dextran	SET-M33 peptide	NP size = 18 nm	**In vitro*:*** Human bronchial epithelial cells RAW264.7 cells **In vitro*:*** BALB/c mice	Microsprayer® nebulizer (suspension)	The M33-nanosystem demonstrated efficacy against *P. aeruginosa* in time-kill kinetic experiments. The retention time of the SET-M33 NPs tripled, significantly enhancing its therapeutic effect in a BALB/c mice model of pulmonary infections	[111]
	Polymer-lipid hybrid (PLGA-lecithin)	Levofloxacin	NP size = 200 − 1000 nm MMAD = 3.3 − 3.7 μm FPF = 38 − 52%	**In vitro*:*** A549 cells **In vivo:** Wistar rats	DPI	Exhibit strong in vitro antimicrobial activity against of *P. aeruginosa.* Nano-aggregates from spray-freeze drying have better aerosolization efficiency than those from spray drying, with a higher emitted dose, finer particle fraction, and lower mass median aerodynamic diameter	[80]
	Porous silicon	Tandem peptide	NP size = 225 nm with pore size range from 13.3 − 14.4 nm	**In vitro*:*** NIH- 3 T3 and Neuro- 2a cells **In vivo:** Mice	Tracheal instillation	Pulmonary delivery of the NPs showed enhanced safety and reduced toxicity, significantly lowering bacterial counts in *P. aeruginosa* infections and leading to improved survival rates compared to untreated mice	[92]
**Pulmonary multidrug-resistant Gram-negative infections (*K. pneumonia*)**	Polymeric and lipid-core micelles	AA139	NP size = 15–20 nm	**In vitro*:*** N/A **In vivo:** Albino rats	Endotracheal aerosolization	In vitro***:*** Both AA139-based NPs exhibited equivalent in vitro antimicrobial activity, comparable to that of free AA139. They demonstrated longer residence times in the lungs compared to free AA139 (approximately 20% longer for AA139-PNP and about 80% longer for AA139-MCL), with reduced toxicity allowing for a higher maximum dose	[79]
**Pulmonary bacterial infection (pneumonia) (*S. aureus* and *MRSA*)**	Magnetic (Fe_3_O_4_)	Vancomycin (VAN)	NP size = 14.7 nm Coating thickness 3–16 nm MMAD = 2.8 − 3.2 µm FPF = 60–70%	**In vitro*:*** WI- 38 cells **In vivo:** Albino rats	DPI	The formulations showed strong activity against *S. aureus* and *MRSA*, with improved biocompatibility, lung tissue localization, and fewer adverse effects than free VAN	[91]
**Pulmonary fungal infection**	Liposomes- phosphatidylcholine-cholesterol-* O*-palmitoyl mannan (OPM) and *O*-polmitoyl pullulan (OPP)	Amphotericin B (AMB)	NP size = 2.27–3.23 μm	**In vitro:** N/A **In vivo:** Albino rats	Pressurized aerosol	Studies on albino rats showed that ligand-anchored liposomal aerosols led to higher lung drug concentrations, with the drug remaining detectable after 24 h	[112]
	Chitosan-stearic acid	Amphotericin B (AMB)	NP size = 101–248 nm MMAD ≤ 6.4 μm FPF = 40% to 54%	**In vitro*:*** Fungal organisms (Candida albicans,Aspergillusniger,Aspergillus fumigatus,Aspergillus flavus, or Cryptococcus neoformans) **In vivo*:*** N/A	Nebulization using Air-jet nebulizer	AMB nanomicelles enhance antifungal activity, making them a promising option for pulmonary delivery	[113]
	Chitosan-PLGA	Voriconazole	NP size = 155–277 nm MMAD = 2.46 − 2.8 μm FPF = 57.5–66.6%	**In vitro*:*** N/A **In vivo*:*** Albino mice	DPI Using (nose-only inhalation chamber)	The coated formulation with chitosan demonstrated longer pulmonary retention and a significantly improved pharmacokinetic profile compared to the non-coated version	[78]
	Polymethacrylic acid	Amphotericin B (AMB)	NP size = 78–94 nm	**In vitro*:*** Human monocyte-derived-macrophages or to A549 cells **In vivo:** BALB/c and C57BL/6 mice	Nebulization	The study shows that non-toxic NPs killed over 99% of Aspergillus, offering proof-of-concept for preventing fungal lung infections	[114]
	Sodium deoxycholate sulfate (SDS)	Amphotericin B (AMB)	NP size = 73.8 nm MMAD = 1.70—1.74 μm FPF = 70—80%	**In vitro*:*** Human lung adenocarcinoma cell lines (A549), human bronchial epithelial cells (Calu- 3) and alveolar macrophage cell lines (ams NR8383) **In vivo:** N/A	Nebulization	The SDS-AMB formulation showed significantly lower toxicity compared to AMB alone in vitro	[115]
**Pulmonary viral infection (COVID- 19)**	SLNPs	Favipiravir (FPV)	NP size = 389–693 nm MMAD = 3 μm FPF = 60.2%	**In vitro*:*** Vero-E6 Cells **In vivo:** N/A	-	Displayed anti-viral activity against SARS-cov- 2 with CC50 and IC50 values of 449.6 µg/ml and 29.9 µg/ml, respectively. Inhalable SLNPs encapsulating FPV show promising activity against coronavirus	[70]
**Vaccine** **(SARS-COV- 2)**	Cationic liposome	SARS-cov- 2 spike protein	NP size = 200 − 1500 nm	**In vitro***:* 3 T3 fibroblasts, CALU- 3 lung cells, primary human fibroblasts (HLF), and rat lung cells (pneumocytes) **In vivo***:* Mice	Intranasal	The vaccine formulation exhibited no toxicity in human bronchial cells or mice in preclinical tests The intranasal liposomal formulation boosted heterologous immunity from a previous Oxford/Astrazeneca® intramuscular vaccination, leading to a stronger response than homologous immunity	[64]
**Vaccine (influenza)**	PLGA	Bovine parainfluenza virus type- 3 (BPI3 V) peptide	NP size = 225 − 330 nm	**In vitro*:*** N/A **In vivo:** BALB/c mice	Intranasal	The antigen-encapsulated NPs triggered a stronger IgG antibody response earlier than the BPI3 V antigen alone	[81]
	Chitosan	Killed swiav H1 N2 (δ-lineage) antigens	NP size = 414.2—571.7 nm	**In vitro*:*** Porcine monocyte-derived dendritic cells, **In vitro**: Pigs	Intranasal mist	**In vitro*:*** Cytokine release in dendritic cells derived from porcine monocytes **In vivo*:*** Enhanced cross-reactive response of T and B lymphocytes	[116]
	Chitosan	Hemagglutinin (HA)	NP size = 351–359 nm	**In vitro*:*** N/A **In vivo**: BALB/c mice	Intranasal	The antigen-encapsulated NPs elicited stronger systemic and mucosal antibody responses compared to the vaccine composed solely of the HA	[82]
**Vaccine (whooping cough)**	Chitosan-dextran	Pertussis toxin (PTX)- IgA)	NP size = 290—330 nm	**In vitro*:*** N/A **In vivo**: BALB/c mice	Intranasal	NPs with IgA and PTX improve nasal mucosa uptake, indicating IgA could be a useful targeting agent for vaccine delivery	[83]
**Vaccine (Pneumonic plague)**	Amino-decorated mesoporous manganese silicate	-	NP size ≈100 nm	**In vitro*:*** N/A **In vivo:** Mice	Intratracheal aerosolization	It enhances antigen presentation and boosts rF1-V10-mediated protection against *Yersinia pestis* infection	[94]

* *MMAD*, Mass Median Aerodynamic Diameter, *FPF*, Fine Particle Fraction, *GSD* Geometric Standard Deviation, *VMD *Volume Median Diameter, *MIC* Minimum Inhibitory Concentration, *P. aeruginosa* *Pseudomonas aeruginosa*,* S. aureus* *Staphylococcus aureus*,* MRSA* *Methicillin resistant Staphylococcus aureus*,* K. pneumonia* *Klebsiella pneumonia*,* S. pneumoniae* *Streptococcus pneumonia*,* M. abscessus* *Mycobacterium abscessus*,* DPI* Dry Powder Inhaler

### Liposomes and lipid carriers for treatment and prevention of pulmonary infectious diseases

Liposomes and lipid NPs are the most studied carriers for delivering various therapeutics to the pulmonary tract via inhalation (see Table 2). Arikayce® and Linhaliq™ are two liposomal-based formulations that have progressed to clinical trials, with Arikayce® successfully reaching commercialization [59]. Arikayce® is the first and currently the only FDA-approved inhalable nanomedicine, approved in 2018, for use as part of a combination therapy for *Mycobacterium avium* complex (MAC) lung disease. It consists of an amikacin in a dipalmitoylphosphatidylcholine (DPPC)—cholesterol liposome suspension designed for nebulization using the Lamira® nebulizer [60, 61]. Linhaliq™ was a liposomal formulation of ciprofloxacin developed by Aradigm, designed for nebulization using a jet nebulizer (PARI LC®). It was developed and clinically tested in two Phase 3 trials (ORBIT) to treat non-cystic fibrosis bronchiectasis (NCFB) associated with *Pseudomonas aeruginosa* infections [62]. It featured a dual-release profile with ciprofloxacin-loaded liposomes for sustained release and free ciprofloxacin for rapid release [63]. However, in 2019, the company withdrew its application for marketing authorization following negative feedback from the FDA [63]. Reports are that Aradigm is currently working on additional submissions for this or other therapeutic indications in the future [63].

Liposomes and other lipid NPs have been extensively studied as potential antimicrobial carriers for the treatment of pulmonary infections via the inhalation route. For example, liposomes have been used to deliver antibiotics such as moxifloxacin, levofloxacin, and rifampicin directly to the lungs, directed towards TB, MAC and other pulmonary bacterial infections. Additionally, these have been investigated for their efficacy in addressing fungal infections (Table 2). Lipid NPs have also shown promise in the treatment and prevention of viral infections, including COVID- 19 [64]. The initial NP sizes ranged from 180 to 380 nm, and they were subsequently formulated and optimized using various techniques to achieve aerodynamic diameters between 1.4 to 5.9 μm. These liposomal formulations enhance drug efficacy by improving macrophage uptake and ensuring higher drug concentrations in lung tissues [65–68]. Solid lipid NPs (SLNPs) have also been utilized to treat pulmonary TB, often loaded with antimicrobials such as rifampicin, either alone or in combination with isoniazid and pyrazinamide. These nanoparticle-based formulations, characterized by aerodynamic diameters of less than 3 µm, have demonstrated improved efficacy by effectively targeting the infection site and reducing dosing frequency [69]. Furthermore, inhalable lipid NP formulations have shown significant promise for delivering antiviral drugs, including those for COVID- 19. For example, SLNPs encapsulating the antiviral drug favipiravir have exhibited potent activity against SARS-CoV- 2, with CC50 and IC50 values of 449.6 µg/ml and 29.9 µg/ml, respectively [70].

Lipid NPs have also been shown to enhance vaccine efficacy by delivering antigens and adjuvants, thereby boosting immune responses for both prophylactic and therapeutic purposes. Given that the respiratory system serves as a key entry point for pathogens, it is an ideal target for vaccine delivery [71]. One notable vaccine employing lipid NPs is the cationic liposome loaded with the SARS-CoV- 2 spike protein. This intranasal formulation enhanced heterologous immunity when used following the Oxford/AstraZeneca® intramuscular vaccination, eliciting a stronger immune response compared to homologous immunity [64]. Another successful example is the dry powder influenza vaccine Inflexal V®*,* which has reached the market. It utilizes liposomal NPs composed mainly of phosphatidylcholine and serves as a cost-effective and efficient vaccine option [72, 73].

### Polymeric NPs for treatment and prevention of pulmonary infectious diseases

Polymeric NPs have also been investigated for treating and preventing pulmonary infectious diseases [48, 74–76]. However, a product based on these particles is yet to reach the market. PLGA, chitosan, alginate and dextran NPs have been loaded with various antibiotics including rifampicin, clarithromycin, tobramycin, and polymyxin B (Table 2). For example, PLGA-poly-L-lysine (PLL) NPs, with sizes ranging from 0.5 to 2 μm, have demonstrated enhanced macrophage uptake of rifampicin, thereby improving its effectiveness against *M. tb* [77]. In the case of fungal infections, chitosan-PLGA NPs encapsulating voriconazole were developed, with a NP size of less than 300 nm. The particles had an MMAD ranging from 2.46 to 2.8 μm, making them well-suited for alveolar desposition. These formulations demonstrated prolonged retention and improved efficacy in pulmonary tissue [78]. Moreover, polymeric micelles and polymer-lipid hybrid formulations with aerodynamic diameters of less than 5 μm have been utilized to combat drug-resistant bacteria by enhancing antibiotic penetration in the lung tissues and maintaining high drug concentrations in lung tissue [79, 80].

Polymeric NPs have shown significant potential in enhancing immune responses and improving vaccine efficacy. PLGA NPs loaded with bovine parainfluenza virus type- 3 (BPI3 V) peptides triggered stronger IgG antibody responses in mice than the antigen alone when administered intranasally [81]. Similarly, chitosan-based NPs encapsulating hemagglutinin (HA) antigens for influenza vaccines demonstrated enhanced systemic and mucosal antibody responses compared to vaccines containing HA alone when administered intranasally [82]. Additionally, whooping cough vaccines utilizing chitosan-dextran NPs have shown improved uptake in the nasal mucosa, suggesting more effective vaccine targeting [83].

Research has evolved from using polymeric NPs primarily for controlled drug release [84] to their application in immunotherapy for infectious diseases [85–89]. Kutscher et al. (2024) reported that rifampicin, when loaded in β-glucan functionalized chitosan–PLGA NPs and administered via the pulmonary route, stimulated the innate immune system by increasing proinflammatory cytokine levels and providing sustained release of rifampicin in vivo for over a week [51]. Similarly, Bekale et al. (2025) demonstrated that biomimetic curdlan–PLGA NPs induced autophagy in *M. tb*-infected macrophages, reduced the bacterial burden in the lungs of H37Rv-infected C57BL/6 mice, and modulated cytokine and chemokine responses [85]. This approach, alongside similar findings using liposomes [90], highlights the potential of nanoparticle-based immunotherapy in combating intracellular pathogens.

### Inorganic NPs for treatment and prevention of pulmonary infectious diseases

Studies which have investigated the efficacy of inorganic NPs for pulmonary infections are relatively few (Table 2). An example is that of magnetic iron oxide (Fe₃O₄) NPs coated with vancomycin, which have been employed against bacterial pneumonia, particularly against *Staphylococcus aureus* and methicillin-resistant *Staphylococcus aureus* (MRSA). These formulations, with an aerodynamic size ranging from 2.8 to 3.2 µm, have demonstrated enhanced lung tissue localization, strong antibacterial activity, improved biocompatibility, and lower toxicity than free vancomycin [91]. Another study investigated the use of porous silicon NPs loaded with tandem peptides to treat *Pseudomonas aeruginosa* infections, showing improved safety, reduced toxicity, significant bacterial reduction, and enhanced survival rates in mouse models [92]. Additionally, gold and silver NPs, known for their intrinsic antimicrobial properties, have been extensively explored [93]. Another type of inorganic NPs is manganese-based NPs, which have been investigated for the prevention of fatal plague (Table 2). These NPs could enhance antigen presentation and increase protection against *Yersinia pestis* infection in mice when administered via intratracheal aerosolization [94].

Although inorganic NPs hold significant promise for pulmonary applications, concerns regarding their bioaccumulation and potential toxicity to healthy cells have limited their widespread adoption and clinical translation. While the production of reactive oxygen species (ROS) and the subsequent ROS-induced oxidative stress are key mechanisms in the efficacy of inorganic NPs, including iron oxide, gold, and silver NPs, this can lead to toxicity in the lungs [95]. However, strategies such as green synthesis, surface modification, control of size and dose, may mitigate these adverse effects [95, 96].

### Approaches in formulation of inhalable nanomedicines

Efficient lung distribution of inhaled NPs has been demonstrated to be influenced by both biological barriers and physicochemical properties, including size, shape, charge, density, and aerodynamic size distribution [54, 55, 117]. Particle size, often expressed by mass median aerodynamic diameter (MMAD) and geometric standard deviation (GSD), is crucial for determining deposition localisation within the lungs [38, 56]. Particles with a MMAD ranging from 3–5 µm primarily deposit in the central airways, while those between 1–3 µm are more suitable for deep lung penetration (alveolar space) [118]. The fraction of particles with an MMAD in the 1–5 µm range, relative to the total amount of powder loaded in a capsule/reservoir or emitted from a dry powder inhalers (DPIs), is defined as the fine particle fraction (FPF). Particles with an MMAD greater than 5 µm tend to deposit in the upper airways, whereas particles smaller than 1 µm have a high probability of being exhaled [119, 120].

Pulmonary administration is achieved using either liquid or solid formulations to generate an aerosol. The three primary medical aerosolization devices are nebulizers, pressurized metered-dose inhalers (pMDIs), and dry powder inhalers (DPIs) [121, 122]. Among these, only nebulizers and DPIs have capacity to deliver the required high doses for low-potency antimicrobials [123, 124]. Selecting an appropriate inhalation device, along with an optimized formulation, is crucial to ensure effective drug delivery to the targeted lung site. Particle size plays a key role in determining the delivery location within the respiratory tract, whether to the upper or lower regions [125]. NPs can be aerosolized in the lungs, either as a suspension or as inhalable dry powder. The latter can be achieved by adsorbing or embedding the NPs onto a carrier, or by agglomerating them into Trojan microparticles or nanoporous microparticles (NPMPs) [126] enabling a respirable particle size range of 1–5 µm [119, 120, 127]. The formulation of these NPs-loaded microparticles for inhalation often relies on spray drying, which is the most commonly used process due to its efficiency and scalability [128]. This technique frequently incorporates deagglomeration enhancers, such as L-leucine or trileucine, to improve dispersibility and ensure the production of respirable particles [129, 130]. Additionally, sugars like lactose, trehalose, or raffinose are commonly included as stabilizers or matrix formers, aiding in the preservation of particle structure and enhancing aerosolization properties [131, 132].

For targeting specific cells, such as alveolar macrophages (AMs) in TB, particle size can aid passive targeting [133], while active targeting is enhanced by modifying NP surfaces with ligands such as curdlan, mannose, or lactose to improve uptake by AMs [86, 134–136].

#### Nebulizers for delivering inhalable nanomedicines

Nebulizers are widely used for pulmonary drug delivery, with three main types: air jet, ultrasonic, and vibrating mesh nebulizers. Air jet nebulizers produce a fine mist using a high-velocity air stream, however, are challenged by low efficiency, noise, and long treatment times [122, 137–139]. Ultrasonic nebulizers offer faster drug delivery and greater lung deposition but may degrade heat-sensitive drugs due to the heat generated during nebulization [140–142]. Vibrating mesh nebulizers, using a perforated mesh to create fine droplets, provide precise delivery and high efficiency, making them promising for clinical applications despite their higher cost and maintenance requirements [139].

Nebulizers offer the advantage of converting liquid formulations into aerosols with minimal processing, preserving the integrity of the nanoformulation. Shear stress is primarily associated with the high linear velocity of jet nebulizers [138], while ultrasonic and vibrating mesh nebulizers may generate heat through cavitation and oscillation [141, 143]. However, in vibrating mesh nebulizers, this effect is minimal due to their single-pass design, preventing prolonged exposure to shear forces or high temperatures [139]. Therefore, careful formulation development and thorough stability assessments are essential for these systems [144, 145]. Additionally, aggregation may occur in highly concentrated nanosuspensions as the distance between particles decreases, increasing inter-particulate forces [146]. This method requires fewer excipients—mainly nanocarriers and suspending agents—that do not significantly impact nebulization, resulting in simpler formulations compared to DPIs [147]. Nanosuspensions are more easily nebulized [141], and are reported to achieve superior lung deposition rates than conventional and micro-formulations due to the aerodynamic sizes of the aggregates [148–150].

Formulations for nebulization are often preferred for preclinical (Table 2) and clinical studies of inhalable nanomedicines, due to their minimal impact on physicochemical properties, support for formulation screening, and effective therapeutic outcomes. The high safety profile and lower incidence of adverse reactions make them ideal for scaling up inhalable nanomedicines. Vibrating mesh nebulizers address several limitations of traditional nebulizers, including challenges with portability, significant medication loss, noise generation, long treatment times, therapeutic instability, and low drug delivery efficiency [122, 138–142]. These advancements enhance patient compliance and improve the effectiveness of formulations [151, 152].

Vibrating mesh nebulizers have led to the development of advanced nebulization technologies, such as Aeroneb® Pro and eFlow®, which perform well in delivering nanoformulations to the lungs [153, 154]. The Lamira® nebulizer system, which utilizes eFlow® technology, is featured in the FDA-approved nanomedicine Arikayce® [60]. Additionally, nebulisers are particularly valuable in the treatment of paediatric patients, who exhibit different airway anatomy and breathing patterns compared to adults. Children are often unable to or find it extremely challenging to use other inhalation devices [155]. Overall, nebulizers, with a particular focus on mesh nebulizers, are considered the most suitable devices for administering inhalable nanomedicines.

#### DPIs for delivering inhalable nanomedicines

Half of the approved inhaled antibacterials are formulated as dry powders, including tobramycin (marketed as TOBI Podhaler) and colistimethate sodium (marketed as Colobreath) [156]. In formulating inhalable nanomedicines using the DPI approach, drying methods play a key role in determining the final product's properties. The goal is to transform NPs into respirable microparticles with a MMAD ranging from 1 to 5 µm, while also addressing challenges such as poor flowability and dispersibility, which are often associated with the cohesive nature and small mass of NPs. Three main drying techniques are used to convert NP suspensions into dry powders composed of microparticles: freeze-drying, spray drying, and spray freeze drying [57, 157]. Freeze-drying may cause NP aggregation, resulting in the formation of excessively large particle and inconsistent powder properties [157, 158], making it unsuitable for the development inhalable formulations. Spray drying is a technique widely used for formulating DPIs due to its ability to produce particles with desired characteristics for pulmonary drug delivery. Spray drying allows for better control over particle size, shape and density but requires careful optimization of experimental parameters to ensure consistent aerodynamic and dispersing properties [57, 159]. The relationship between spray-drying parameters and the in vitro aerosol performance of inhalable nanomedicines is formulation-specific [160]. For instance, Wan et al*.* observed no clear correlation between spray-dryer feed rate and either the geometric median diameter of the particles or the in vitro aerosol performance of spray-dried itraconazole nanoagglomerate microparticles. This finding contrasts with the typical expectation that a higher feed rate would produce larger droplet and particle sizes [160]. Interestingly, spray drying can be combined with other techniques to enhance the properties of DPI formulations. For example, the use of cocrystallisation during spray drying can improve the humidity resistance of drugs [161]. Additionally, spray drying can be used to produce NP agglomerates which can enhance the delivery of poorly water-soluble drugs and achieve modified drug release [162]. However, spray drying has limitations, particularly for heat-sensitive compounds and materials with low melting points, such as polycaprolactone (PCL) polymer, which is commonly used as a nanocarrier [160]. Among the three processes mentioned earlier, spray drying stands out as the most scalable [128].

Spray freeze drying is an advanced technique capable of protecting heat-sensitive materials and often demonstrates superior aerodynamic performance compared to spray drying [80, 163–165]. This advantage is largely due to the high particle porosity resulting from water removal during the sublimation of frozen droplets [57]. However, debate over the aqueous dispersibility of spray freeze drying compared to spray drying remains unresolved. While several studies suggest that spray freeze drying provides superior dispersibility [80, 163, 164], Yu et al. reported opposite findings, attributing this to the formation of ice crystals during the freezing process [165]. Spray freeze drying also faces challenges such as low production rates due to the prolonged lyophilization process and difficulties in scaling up for manufacturing. As a result, despite its advantages, it is less frequently studied for the production of inhalable nanomedicines compared to spray drying.

Furthermore, strategies for formulating inhalable nanomedicines using DPIs, such as nano-embedded microparticles and nanoparticle-carrier systems, often require large amounts of excipients, resulting in low drug loading and inconsistent lung deposition [166, 167]. Nano-agglomerate microparticles can incorporate protective agents, such as oses (lactose, raffinose, trehalose) and sugar derivatives (mannitol), amino-acids (glycine, proline), to shield NPs from stress during spray drying and storage. These agents stabilize the NPs through mechanisms such as hydrogen bonding, formation of a vitrified (glassy) matrix, and suppression of crystallization, while also mitigating thermal and mechanical stresses. However, use of these agents can negatively affect powder flowability, aerosol performance, and drug loading within the microparticles, making their careful selection and concentration critical for achieving optimal delivery. [57]. Additionally, ensuring batch-to-batch consistency and stability during industrial-scale production remains a significant challenge [168, 169].

## Clinical trials of inhalable nanomedicine for infectious diseases

**Fig. 1:**
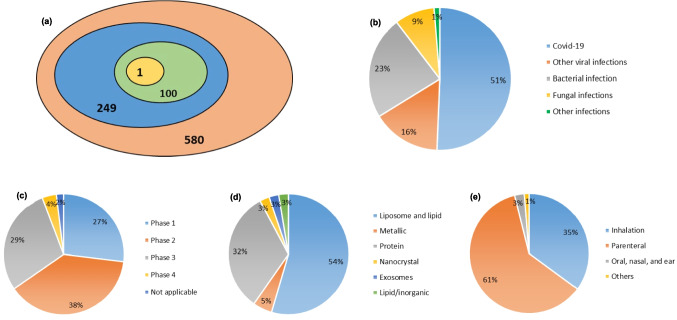
Overview of nanomedicines for pulmonary infectious diseases: (**a**) Logic diagram illustrating the total number of registered clinical trials involving nanomedicines (580), those with completed results (249), FDA approvals as of 2024 (100), and FDA approvals for infectious diseases via the pulmonary route (1). Panels (**b**), (**c**), (**d**), and (**e**) show nanomedicines currently in clinical trials, including (**b**) types of infectious diseases, (**c**) development status, (**d**) types of NPs, and (**e**) routes of delivery. Data were collected and analyzed from ClinicalTrials.gov

Our analysis of the clinical status of nanomedicines for pulmonary infections, based on data from PubMed, ClinicalTrials.gov and FDA databases [58, 170, 171], revealed that by December 2024, approximately 7,115 publications had addressed the clinical translation of nanomedicines. Of these studies, 580 had progressed to clinical trials or other stages, including 77 trials specifically focused on pulmonary infections. These efforts have resulted in over 100 FDA approvals, including nine for infectious diseases. However, only one of these, Arikayce®, has been approved for inhalation administration (Fig. 1a) [172–175].

The involvement of nanomedicines in trials for pulmonary infectious diseases has significantly increased, especially since the onset of the COVID- 19 pandemic. Viral infections account for about 67% of these trials, with COVID- 19 studies representing approximately 51% of that total (Fig. 1b). Most nanomedicines for pulmonary infections are currently in Phase I (27%), Phase II (38%), and Phase III (29%) trials (Fig. 1c). Various types of NPs are being explored, with a particular focus on lipid-based NPs (54%) and protein NPs (32%) (Fig. 1d). The parenteral route remains the most common method of administration in clinical trials (61%), followed by inhalation (35%) (Fig. 1e). Table 3 provides a summary of the development status of inhalable nanomedicines for pulmonary infectious diseases, including those already approved for marketing and those in ongoing clinical trials.

**Table 3:** Clinical development of inhalable nanomedicines for pulmonary infectious diseases

No	Trade name	Inhalation technique	Therapeutic agent/NPs	Indication	Approval/Clinical trial status	Sponsor organization
1	Arikayce®	Inhalation- nebulizer	Liposomal/amikacin	Non-tuberculous mycobacterial lung infection caused to *MAC*	Marketed (FDA, 2018), (EMA, 2020) [176]	Insmed Incorporated
2	Amikacin Liposome Inhalation Suspension (ALIS)	Inhalation- nebulizer	Liposomal/amikacin	Non-tuberculous mycobacterial lung infection due to *MAC*	NCT04677569 (phase III)	Insmed Incorporated
3	Arikace®	Inhalation- nebulizer	Liposomal/amikacin	Patients with cystic fibrosis with bacterial infection *P. aeruginosa*	NCT00558844 (phase I/II)	Insmed Incorporated
4	Arikayce®	Inhalation- nebulizer	Liposomal/amikacin	Patients with cystic fibrosis with bacterial infection *P. aeruginosa*	NCT05999942 (phase I)	Insmed Incorporated
5	Arikayce®	Inhalation- nebulizer	Liposomal/amikacin	Chronic *P. aeruginosa* infections in patients with cystic fibrosis	NCT01315678 (phase III)	Insmed Incorporated
6	Arikayce®	Inhalation- nebulizer	Liposomal/amikacin	Patients with bronchiectasis and Chronic Infection Due to *P. aeruginosa*	NCT00775138 (phase II)	Insmed Incorporated
7	Arikayce®	Inhalation- nebulizer	Liposomal/amikacin	Patients with cystic fibrosis with *P. aeruginosa* infection	NCT00777296 (phase I/II)	Insmed Incorporated
8	Arikayce®	Inhalation- nebulizer	Liposomal/amikacin	Patients with cystic fibrosis with *P. aeruginosa* infection	NCT01315691 (phase III)	Insmed Incorporated
9	COVID- 19EXO	Inhalation- nebulizer	Exosome	COVID- 19 Associated Pneumonia	NCT04491240 (phase I/II)	Samara Regional Medical Center Dinasty
10	COVID- 19EXO2	Inhalation- nebulizer	Exosome	COVID- 19 Associated Pneumonia	NCT04602442 (phase II)	Olga Tyumina
11	Liposomal Amikacin for Inhalation (LAI)	Inhalation- nebulizer	Liposomal/amikacin	Non-tuberculous mycobacterial lung infection caused to *MAC*	NCT02344004 (phase III)	Insmed Incorporated
12	Liposomal Amikacin for Inhalation (LAI)	Inhalation- nebulizer	Liposomal/amikacin	*P. aeruginosa* infections in patients with cystic fibrosis	NCT01316276 (phase III)	Insmed Incorporated
13	Liposomal Amikacin for Inhalation (LAI)	Inhalation- nebulizer	Liposomal/amikacin	non-tuberculous mycobacterium (NTM) lung infections due to *MAC*	NCT02628600 (phase III)	Insmed Incorporated
14	Liposomal Amikacin for Inhalation (LAI)	Inhalation- nebulizer	Liposomal/amikacin	*Mycobacterium. abscessus* lung infection	NCT03038178 (phase II)	Kevin Winthrop
15	Liposomal Amikacin for Inhalation (LAI)	Inhalation- nebulizer	Liposomal/amikacin	Non-tuberculous Mycobacteria infection	NCT01315236 (phase II)	Insmed Incorporated
16	Liposomal Amikacin for Inhalation (LAI)	Inhalation- nebulizer	Liposomal/amikacin	Patients with cystic fibrosis with bacterial infection *P. aeruginosa* infection	NCT03905642 (phase II)	Insmed Incorporated
17	Liposomal Amikacin for Inhalation (LAI)	Inhalation- nebulizer	Liposomal/amikacin	Treatment of *Mycobacterium xenopi* lung disease	NCT06585020 (phase II)	Centre Hospitalier Universitaire, Amiens
18	Pulmaquin®	Inhalation- nebulizer	Liposomal/ciprofloxacin	Non-cystic fibrosis bronchiectasis	NCT01515007 (phase III)	Aradigm Corporation
19	Pulmaquin®	Inhalation- nebulizer	Liposomal/ciprofloxacin	Non-cystic fibrosis bronchiectasis	NCT02104245 (phase III)	Aradigm Corporation
20	Pulmaquin®	Inhalation- nebulizer	Liposomal/ciprofloxacin	Non-cystic fibrosis bronchiectasis	NCT01515007 (phase III)	Aradigm Corporation
21	Ambineb	Inhalation- nebulizer	Liposomal/Amphotericin B	Pulmonary Aspergillosis	NCT00391014 (phase II)	PETHEMA Foundation
22	Liposomal amphotericin B (ALN)	Inhalation- nebulizer	Liposomal/Amphotericin B	Pulmonary Aspergillosis	NCT04267497 (phase II)	Fundacion para la Investigacion Biomedica del Hospital Universitario Ramon y Cajal
23	Liposomal amphotericin B (ALN)	Inhalation- nebulizer	Liposomal/Amphotericin B	Pulmonary Aspergillosis	NCT00263315 (phase II/III)	Erasmus Medical Center
24	AmBisome®	Inhalation- nebulizer	Liposomal/Amphotericin B	Pulmonary Mucormycosis	NCT04502381 (Phase II)	Post Graduate Institute of Medical Education and Research, Chandigarh
25	Abelcet ®	Inhalation- nebulizer	Liposomal/Amphotericin B	Prevention pulmonary fungal infection	NCT00177684 (phase III)	University of Pittsburgh
26	AmBisome®	Inhalation- nebulizer	Liposomal/Amphotericin B	Pulmonary Aspergillosis	NCT03656081 (phase III)	Poitiers University Hospital
27	AmBisome®	Inhalation- nebulizer	Liposomal/Amphotericin B	Pulmonary Aspergillosis	NCT03327727 (phase II)	Vical
28	Remdesivir (GS- 5734™)	Inhalation- nebulizer	Nanocrystal-Remdesivir (GS- 5734™)	Covid19, Severe Acute Respiratory Syndrome (SARS) Pneumonia	NCT04480333 (Phase I)	Biomed Industries, Inc

*For additional information on the status of clinical trials, please visit https://clinicaltrials.gov

## Conclusion and future directions

There is significant investigation of NPs for treatment and prevention of pulmonary disease with liposomes and lipid NPs being the most investigated. At least one product, Arikayce® has marketing authorization from the US FDA. Despite these studies and advancements, notable gap persists in translational research, with challenges in attaining the target product profile, availability of appropriate in vivo disease models, scale-up, and market related questions, likely hindering research translation to the clinic. Optimizing device selection and formulation parameters is also essential in the development of inhalable nanoformulations to ensure effective inhalation and lung deposition and patient adherence. The use of pMDIs for delivering nanoformulations requires further investigation, particularly regarding propellant-related challenges. It is crucial to explore strategies that minimize the impact on NP stability while improving drug deposition rates in the lungs [122]. DPIs also require further optimization, particularly in terms of formulation components and drying process parameters [157, 177]. Future advancements in technologies such as nano-spray drying and supercritical fluid drying [128] could improve the suitability of DPIs for nanoformulations. Vibrating mesh nebulizers, including eFlow® and AKITA® APIXNEB systems, show promise due to their low drug loss, reduced shear stress, and electronic controls that adjust flow rate, output, and aerosol duration for enhanced delivery [139]. However, portability of these systems is an issue.

Inhalable nanoformulations require validation for safety and efficacy in relevant preclinical models—including in vitro, ex vivo, and in vivo methods—to predict human responses prior to clinical trials [178, 179]. While advances have been made in animal models for diseases like cystic fibrosis, there remains a need for models that more accurately reflect chronic lung infectious diseases. Despite their limitations, in vitro models offer valuable insights for optimizing formulation design [180]. The FDA Modernization Act 2.0 allows alternatives to animal testing, including cell-based assays (such as organoids and organs-on-chips) and advanced artificial intelligence (AI) methods [181, 182]. Additionally, computational approaches such as dosimetry models and physiologically based pharmacokinetic models can predict the deposition and clearance of inhaled nanomedicines in the respiratory tract, aiding in understanding particle distribution and movement [183–188].

Charting the way forward, we see promise from host directed therapies for these pulmonary diseases [85, 86, 189]. In the context of growing microbial resistance, host-directed therapies offer a compelling solution, providing potential for effective treatment including against drug resistant strains. There is also potential to combine the action of inherently antimicrobial NPs and/or their components with antibiotics to obtain synergistic activity against the pathogens, which could potentially mitigate the occurrence of drug resistant strains. Our review of current studies on NPs in pulmonary infectious diseases highlights a lack of clinical studies on formulations beyond liposomes, such as polymers. The reasons why polymeric NPs are not advancing to clinical trials and reaching the market remain unclear. Therefore, further investigation and translation of these systems is recommended. Additionally, there is a need for further pharmacokinetic studies, including profiling NP biodistribution and intracellular drug distribution within the lungs and cells in relevant models, to better understand the potential of these NP systems and optimize them for clinical translation.

## Data Availability

All tables and figures in this review are based on previously published research, which is cited throughout. Additional details are available from the corresponding author upon request.
